# Impact of *KRAS* Mutation on Recurrence-Free Survival in Colon Cancer Patients Undergoing Cytoreduction with Hyperthermic Intraperitoneal Chemotherapy in a Predominantly Hispanic Population

**DOI:** 10.3390/cancers18142301

**Published:** 2026-07-17

**Authors:** Alexandra E. Hernandez, Peter A. Borowsky, Charles J. Cash, Mariaugusta Montealegre, Kimberly P. Gomez, Daniel Noe, Sina Yadegarynia, Agustin Pimentel, Heidi Bahna, Mecker G. Möller

**Affiliations:** 1 Department of Surgery, University of Miami Miller School of Medicine, Miami, FL 33136, USA; 2 Sylvester Comprehensive Cancer Center, University of Miami, Miami, FL 33136, USA; 3 University of Miami Miller School of Medicine, Miami, FL 33136, USA; 4 HCA Florida JFK Hospital, Atlantis, FL 33462, USA; 5 Division of Hematology-Oncology, Department of Medicine, University of Miami Miller School of Medicine, Miami, FL 33136, USA; 6 Section of Surgical Oncology, Department of Surgery, University of Chicago Pritzker School of Medicine, Chicago, IL 60637, USA

**Keywords:** KRAS mutation, colon cancer, HIPEC, oncologic outcomes, cancer survival

## Abstract

**Introduction**: For colon cancer (CC), *KRAS* mutations are associated with worse outcomes. Cytoreductive surgery (CRS) with hyperthermic intraperitoneal chemotherapy (HIPEC) for advanced-stage CC may proffer some survival benefits for select patients. We sought to evaluate postoperative morbidity and mortality among mutant and wildtype *KRAS* patients undergoing CRS/HIPEC for advanced CC. **Methods**: We identified 80 patients with CC from an institutional HIPEC database. Patients were categorized based on the presence (mut*KRAS*) or absence (wildtype) of *KRAS* mutations. Patient characteristics, tumor factors, treatment, and clinical outcomes were compared between groups. Postoperative morbidity was measured by Clavien–Dindo Classification (CDC). Kaplan–Meier survival analysis evaluated recurrence-free survival (RFS). **Results**: Overall, 52 (65%) mut*KRAS* were identified. Patient and tumor characteristics were similar among groups. While not significant, the wildtype group had a lower median (IQR) preoperative PCI score of 7 (3, 12) than the mut*KRAS* group, whose score was 11 (5, 21) (*p* = 0.057). The mutKRAS group had a higher rate of CDC grade 3 or more (23.1%) compared to the wildtype group (7.1%) (*p* = 0.015). Despite this, there were no significant differences in length of stay. Moreover, RFS between the mut*KRAS* and wildtype groups did not differ significantly (*p* = 0.47). **Conclusions**: Contrary to the typical association of *KRAS* mutations with poorer survival in CC, our findings suggest that patients with this mutation have an RFS comparable to those without it when treated with CRS/HIPEC. This may imply that CRS/HIPEC offers therapeutic benefits for *KRAS*-mutant CC patients, potentially mitigating the usually adverse prognosis associated with this mutation.

## Simple Summary

In advanced colon cancer, *KRAS* mutations (mut*KRAS*) typically predict poorer outcomes. After evaluating 80 patients undergoing CRS/HIPEC, we found mut*KRAS* had higher postoperative complications but similar recurrence-free survival (*p* = 0.47). CRS/HIPEC may mitigate *KRAS*-associated prognosis disparities.

## 1. Introduction

Colorectal cancer (CRC) is a molecularly heterogeneous disease accounting for the second most cancer-related deaths [1,2]. An estimated 17% of patients with metastatic CRC (mCRC) have peritoneal carcinomatosis, with 2% having isolated peritoneal metastasis [3]. Patients with peritoneal metastases generally have a shorter recurrence-free survival (RFS) and overall survival (OS) than those without peritoneal involvement [4]. Among CRCs, tumors with *KRAS* mutations are generally associated with increased tumor aggressiveness, treatment resistance, and decreased survival [5]. While *KRAS* has been extensively studied in mCRC, recent trials have also looked at its impact on Stage II and III CRC, showing consistent adverse prognostic effects on RFS [6]. While newer drugs are beginning to be tested for specific *KRAS* mutations, such as G12C *KRAS* mutations, there is still much to be done to treat mut*KRAS* CRC and improve outcomes [7].

Although still controversial, the role of cytoreductive surgery (CRS) with the addition of hyperthermic intraperitoneal chemotherapy (HIPEC) for advanced-stage CC with synchronous peritoneal metastasis may proffer some survival benefit. Clinical acceptance of CRS/HIPEC in patients with isolated peritoneal metastasis from CRC origin is based on a Dutch monocentric randomized controlled trial (RCT) published in 2003 [8]. This was the first positive RCT to demonstrate benefit from CRS/HIPEC, with mitomycin C playing a pivotal role in the development of the therapeutic strategy against CRC with synchronous peritoneal metastasis. In contrast, recent trials such as the PRODIGE-7 trial, which compared the added effect of low-dose, short-perfusion-time oxaliplatin-based CRS/HIPEC against CRS alone demonstrated no additional benefit, with an increase in long-term morbidity in the HIPEC group [9]. These findings called into question the addition of oxaliplatin-based HIPEC to CRS for high-risk CRC patients with synchronous peritoneal metastasis; however, those findings should not be extrapolated to mitomycin-based HIPEC [10].

Several recent RCTs have investigated the role of CRS/HIPEC in high-risk CRC in both the prophylactic and adjuvant setting [11]. The COLOPEC trial compared adjuvant chemotherapy versus adjuvant HIPEC followed by chemotherapy and failed to show a significant reduction in 18-month peritoneal metastases-free survival with incorporation of adjuvant HIPEC with oxaliplatin among patients deemed high-risk for peritoneal carcinomatosis (T4 disease or perforated colon cancer) [12]. In the PROPHYLOCHIP trial, enrollment was limited to patients at elevated risk for peritoneal metastasis who remained free of recurrence following six months of adjuvant chemotherapy [13]. Participants were randomized to either exploratory laparotomy with oxaliplatin-based HIPEC or a surveillance strategy, with no difference in 3-year disease-free survival (DFS) observed between the two groups [13]. The HIPECT4 trial is the first RCT to demonstrate clinical benefit of HIPEC in addition to CRS in patients with high-risk T4 CRC [11,14]. This trial sought to compare CRS versus concurrent CRS/HIPEC with mitomycin C in randomized patients with locally advanced colon cancer. Patients treated with CRS/HIPEC showed a significant increase in 3-year locoregional control of disease, with no increase in adverse events despite not demonstrating differences in DFS or OS [14]. This was the first RCT investigating HIPEC in CRS using mitomycin C instead of oxaliplatin, a chemotherapy agent that demonstrates improved efficacy in CRS/HIPEC for CRC [15]. These findings have returned HIPEC into the spotlight for discussion on the management of CRC and highlighted the importance of careful patient selection [16].

Although CRC with a *KRAS* mutation is considered higher risk, this was not considered in patient selection or controlled for in the aforementioned RCTs. Moreover, relatively few studies have described or evaluated outcomes in patients with mutKRAS versus wildtype CC undergoing CRS/HIPEC for advanced CC. To fill this critical gap, our study sought to assess the impact of the *KRAS* mutation on morbidity and mortality associated with CRS/HIPEC in patients with CC. We hypothesized that mutKRAS patients would have shorter survival times and worse postoperative morbidity compared to wildtype *KRAS* patients due to the known aggressiveness of these tumors.

## 2. Methods

### 2.1. Study Population

**Figure 1:**
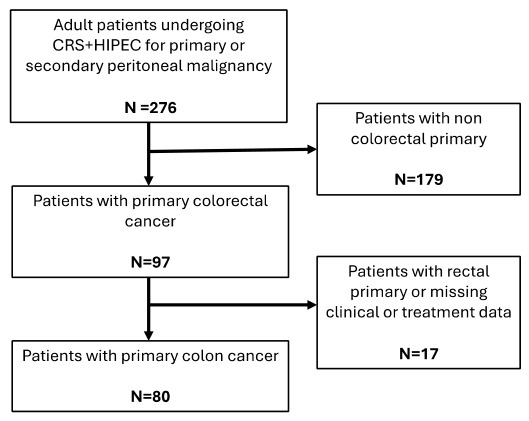
Study flow diagram.

Patients who were scheduled to undergo cytoreductive surgery with hyperthermic intraperitoneal chemotherapy (CRS/HIPEC) for peritoneal surface malignancies between January 2011 and August 2023 at our tertiary center were enrolled in this prospective cohort study. Of the 276 patients undergoing CRS/HIPEC who were enrolled, we included those with a proven colon primary on pathology who had complete clinical information regarding their HIPEC treatment. We chose to exclude those with rectal cancer primary due to the small subgroup (n = 5) and differences in treatment and survival compared to colon primary. We identified 80 patients with CC that met the inclusion criteria (Figure 1). Informed consent to participate in this study was waived for retrospective chart review due to minimal risk. The study protocol was approved by the local Institutional Review Board at the University of Miami (IRB 2013710).

All patients were reviewed by a multidisciplinary tumor board prior to selection for CRS/HIPEC. Following exploratory laparotomy and Peritoneal Cancer Index (PCI) assessment, complete cytoreductive surgery was performed to remove all visible tumors. Procedures included, as indicated, omentectomies, peritonectomies, bowel resection(s), splenectomy, liver capsulectomy and/or wedge resection(s), resection of abdominal wall and bladder implants, and total abdominal hysterectomy with bilateral salpingo-oophorectomy. The goal was to obtain a completeness of cytoreduction score of CC-0 or CC-1. If unsuccessful, a CC-2 score was obtained and considered a palliative resection. HIPEC was then administered via the closed-abdomen technique with two pelvic inflow cannulas and a single upper abdominal outflow cannula, maintaining an intra-abdominal temperature of 42 °C for 90 min. Mitomycin C was delivered in three weight-based doses: 17.5 mg/m^2^ at time 0, followed by 8.8 mg/m^2^ at 30 and 60 min. After perfusion, the abdomen was drained and irrigated, and reconstruction was performed as indicated.

### 2.2. Variables

Patient characteristics included age, sex, and self-identified race and ethnicity. Clinical, tumor and treatment characteristics were gathered by electronic medical record review. Clinical characteristics collected included stage at diagnosis, presenting symptoms, preoperative Eastern Cooperative Oncology Group (ECOG) status, number of surgeries before HIPEC, and whether ascites was drained. Tumor characteristics included grade, histology, mutation status, and preoperative Peritoneal Cancer Index (PCI) score. Treatment characteristics included receipt of neoadjuvant or adjuvant chemotherapy, any other oncologic surgery, radiation, chemotherapeutic agents used in HIPEC, residual tumor classification, and PCI score post-exploration.

### 2.3. Exposure and Outcome

Our exposure in this study was the presence of *KRAS* mutations (mut*KRAS*). Patients were categorized based on the presence (mut*KRAS*) or absence (wildtype) of *KRAS* mutations. This data was collected from tumor tissue pathology reports from electronic medical records. Our primary outcome was RFS. Occurrence and dates of recurrence and death were collected from electronic medical records. RFS time was calculated from the date of surgery to the date of recurrence or death or the last clinical follow-up where the patient was known to be alive. Our secondary outcomes were postoperative outcomes including postoperative morbidity measured by the Clavien–Dindo Classification (CDC) and hospital and intensive care unit (ICU) length of stay (LOS).

### 2.4. Statistical Analysis

We calculated descriptive statistics for all covariates as frequencies for categorical variables and reported the mean ± standard deviation (SD) or median (interquartile range (IQR)) for continuous variables as appropriate. Pearson’s Chi-Square tests and Analysis of Variance (ANOVA) were used, as appropriate, for intergroup bivariate analysis. Standard parametric and nonparametric tests were used to compare continuous variables. The normality of continuous variables was verified using the Shapiro–Wilk test. Bonferroni correction was applied for multiple comparisons. Kaplan–Meier survival analysis evaluated RFS between groups. An alpha level of significance of 0.05 was used for the study. IBM Statistical Product and Service Solutions (SPSS) version 28 was used to conduct statistical analysis.

## 3. Results

### 3.1. Patient and Tumor Characteristics

A total of 276 patients undergoing CRS/HIPEC were identified from our prospective institutional registry. From this cohort, 80 patients were identified as undergoing CRS/HIPEC for treatment of CC primary malignancy. Of these, 52 (65%) were identified as mut*KRAS* and 28 (35%) were wildtype. The mean overall age was 51 (11) years, and when stratified, was 52 (12) years for the mut*KRAS* group and 50 (11) years for the wildtype group. The majority of the cohort (n = 76; 95%) presented with Stage III or IV disease. Those that presented with Stage II disease either had a T4 tumor or were found to have more advanced disease upon surgical exploration. Baseline patient and tumor characteristics between mutant and wildtype *KRAS* groups were not significantly different, yielding comparable groups for analysis (Table 1).

### 3.2. Treatment Characteristics

The median time to CRS/HIPEC was 23.03 (13.95–42.55) months for mut*KRAS* and 18.85 (11.47–39.93) months for wildtype *KRAS* and did not differ between groups (*p* = 0.478). There was no difference in the proportion of patients treated with surgery before CRS/HIPEC (51.9% vs. 64.3%; *p* = 0.288), neoadjuvant chemotherapy before CRS/HIPEC (53.8% vs. 71.4%; *p* = 0.126), or surgery and chemotherapy before CRS/HIPEC (51.9% vs. 64.3%; *p* = 0.288). The median PCI at the time of surgical exploration was higher for mut*KRAS* patients (11, 3–12) compared to wildtype *KRAS* patients (7, 5–21), although this was not statistically significant (*p* = 0.057) (Table 2).

### 3.3. Perioperative Outcomes

The mut*KRAS* group had fewer patients achieve an R0 resection (68.8% vs. 84.6%), although this was not significant. Complication rates were overall higher in the mutKRAS group (*p* = 0.015). Median intensive care unit LOS (3 [2–5] vs. 3 [2–6] days; *p* = 0.803) and overall hospital LOS (10 [7–15] vs. 9 [7–12] days; *p* = 0.256) did not differ between mut*KRAS* and wildtype *KRAS* groups (Table 2).

### 3.4. Survival Analysis

**Figure 2:**
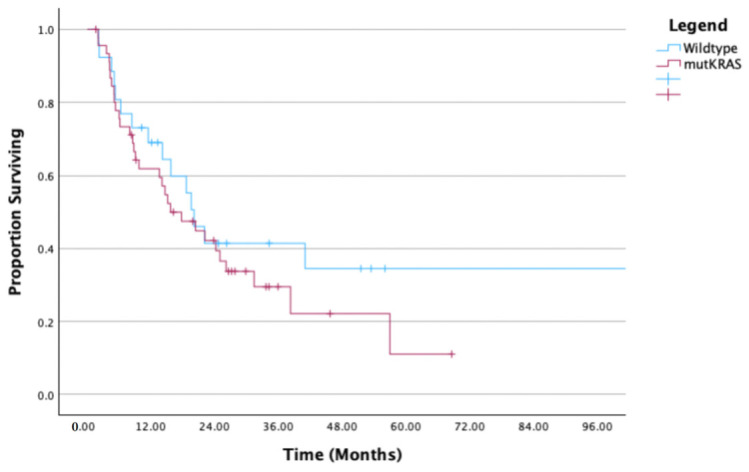
Kaplan–Meier survival curves for mut*KRAS* and wildtype colon cancer patients undergoing HIPEC.

Seventy-two patients with complete postoperative follow-up data were included in the survival analysis. During the study period, 46 recurrence or death events occurred, including 31 in the KRAS-mutant cohort and 15 in the wildtype cohort. The median (IQR) follow-up time for the entire cohort was 51.6 months (35.2–77.2). The median follow-up time did not differ between the mut*KRAS* (51.2, 37.9–71.5) and wildtype *KRAS* (70.3, 34.5–85.6) groups (*p* = 0.219). Median recurrence-free survival was 15.7 months (95% CI, 8.0–23.3) among patients with KRAS-mutant tumors compared with 20.1 months (95% CI, 15.0–25.2) in the wildtype cohort. Although recurrence-free survival was numerically shorter in the KRAS-mutant group, this difference was not statistically significant (log-rank *p* = 0.337) (Figure 2).

## 4. Discussion

Our study found that despite mutant-type *KRAS* patients having higher rates of postoperative complications, there was no significant difference in RFS between groups over 96 months. Additionally, we found no differences in ICU and overall hospital LOS and extent of resection. These findings contradict our original hypothesis and are encouraging given the usually poorer prognosis in mutant-type *KRAS* tumors. CRS/HIPEC in CRC has yet to show a promising survival benefit in many studies, and careful patient selection is an important factor. The findings from this study suggest that mutant-type *KRAS* may be an important factor to consider in optimizing patient selection for CRS/HIPEC.

While postoperative complications were more common among patients with KRAS-mutant tumors, this finding should be interpreted cautiously. Patients in the mutant KRAS cohort also demonstrated a higher median PCI, underwent more extensive cytoreduction, as reflected by a greater median number of organs debulked and peritonectomies, and were somewhat less likely to achieve complete cytoreduction, although none of these differences reached statistical significance. These trends suggest that the increased postoperative morbidity may reflect greater disease burden and operative complexity rather than the independent effect of KRAS mutation itself. Because multivariable adjustment was not feasible in this cohort, we cannot determine whether KRAS mutation independently contributes to postoperative complications, and this association warrants further investigation in larger studies.

Although the impact of *KRAS* mutations on outcomes following CRS/HIPEC for CC remains a scarcely studied field, the results presented here support existing literature. A study by Graf et al. of 110 patients undergoing CRS/HIPEC for colorectal and appendiceal peritoneal metastases found no difference in OS between patients with mutant versus wildtype *KRAS* [17]. In fact, the authors found a non-significant longer survival time in mutant-type *KRAS* patients. Although the study did not separate appendiceal versus colorectal primary tumors, only 13 (11.8%) of the 110 cases were of appendiceal origin. Our study builds upon these findings by only including those of pathologically confirmed colon origin. Another study on CRC by Massalou et al. similarly found no difference in median length of OS and DFS between patients with mutant and wildtype *KRAS* [18]. However, this study took place over 10 years earlier, resulting in a large amount of missing data on mutation status.

Other studies, meanwhile, present conflicting results and show that mutant-type *KRAS* profiles are associated with impaired survival for patients with CRC undergoing CRS/HIPEC. For example, one study by Schneider et al. of 524 patients undergoing CRS/HIPEC for CRC with peritoneal metastases found that mutant-type *KRAS* was associated with a 46% higher likelihood of impaired survival (median cancer-specific survival of 38 months for mutant-type KRAS versus 52 months for wildtype *KRAS*) [19]. In addition, mutant-type *KRAS* was associated with a 42% higher likelihood of shorter RFS, which in the setting of the study was equivalent to a 4-month shorter RFS [19]. While these results are contradictory to those of the present study, it should be noted that only 29.5% of patients in the study had mutant-type *KRAS*, whereas in the present study 65% of patients had mutant-type *KRAS*. Furthermore, the present study differs in that 93% of patients had stage T3 or T4 disease compared to only 63% in the Schneider study; in addition, the median PCI in the present study was higher than that in the Schneider study. The results of our study, which included a higher proportion of patients with mutant-type *KRAS* and a higher proportion of stage T3 or T4 disease, are therefore encouraging.

As alluded to previously, metastatic CRC often carries a poor prognosis, with as many as 17%, and likely even more who are undiagnosed, of patients diagnosed with mCRC also having peritoneal carcinomatosis. Treatment of Stage IV mCRC is mostly palliative, with best practice supportive care offering a dismal 9-month estimated median survival [20]. The addition of 5-fluorouracil/leucovorin plus oxaliplatin therapy has shown promising improvement, with estimated survival increased to 14–19 months; however, prognosis overall remains poor [20]. The use of CRS/HIPEC as a means of treatment for advanced-stage CRC has frequently been debated; however, recent RCTs, including the HIPECT4 trial, have reestablished its clinical benefit [14]. This trial, for example, showed that high-risk T4 CRC patients treated with CRS/HIPEC with mitomycin C had an increased 3-year locoregional disease control while showing no differences in DFS or OS compared to those treated with CRS alone [21]. Mitomycin C has recently been documented as a cornerstone in HIPEC therapy due to its intrinsic activity against colorectal cancer cells, heat synergism, and large AUC intraperitoneal to intravenous ratio [21]. More than 96% of patients in the present study received mitomycin C therapy, which may have contributed to the observed results.

It should also be noted that while many of the initial studies assessing the efficacy of CRS/HIPEC indicated no beneficial impact of CRS/HIPEC for treatment of advanced CC patients, many of these may not have optimally selected patients for this therapy. Patient selection for CRS/HIPEC is becoming increasingly important, and choosing those most likely to benefit from this extensive treatment modality may impact results. For example, in the PRODIGE7 trial, which randomized mCRC patients to receive either CRS/HIPEC or CRS alone and found no difference in survival plus an increased long-term morbidity for patients treated with HIPEC, 25% of the patients had a PCI greater than 15 [9]. This has previously been reported as a threshold for which CRS/HIPEC is not beneficial [22,23]. Furthermore, subgroup analysis of patients from the study found that there was indeed a survival benefit of CRS/HIPEC for patients with an intermediate extent of peritoneal disease (PCI 11–15) [9]. Other studies, meanwhile, such as those by Schneider et al., have directly assessed the impact of prognostic scores to best stratify patients prior to CRS/HIPEC. Schnieder et al. developed a BIOSCOPE score (BIOlogical Score of COlorectal PEritoneal metastasis), consisting of a PCI score, nodal status, tumor grade, and *KRAS* mutation status, and found that the novel score adequately predicted patient prognosis prior to and with CRS/HIPEC [19]. Our findings may help contribute to future prognostic scores as well. Other prognostic calculators such as the colorectal peritoneal metastases prognostic surgical score (COMPASS), the COloREctal-PC (COREP) score, the peritoneal surface disease severity score (PSDSS), the prognostic score (PS), and the CEA/PCI ratio exist to aid in optimal patient selection. However, none of these scores include mutational statuses, which could further aid in optimizing patients for HIPEC [24]. These aforementioned findings, along with those we present here, imply that *KRAS* mutations are not poor prognostic indicators for inferior survival in mCRC patients with peritoneal metastases undergoing CRS/HIPEC, suggesting that optimizing patient selection is an important factor in improving outcomes after CRS/HIPEC.

Notably, our cohort was predominantly composed of ***Hispanic patients***, a population historically underrepresented in genomic and national clinical databases. This demographic composition may account for the higher rate of KRAS mutations observed in our study compared to prior reports in predominantly White, non-Hispanic cohorts. Indeed, a recent genomic study demonstrated significant differences in mutation rates of commonly altered genes—including APC, TP53, KRAS, GNAS, and NOTCH—in Hispanic patients with metastatic colorectal cancer compared to non-Hispanic White and Black patients [25]. These findings underscore the importance of including diverse patient populations in oncologic research, and our study contributes a meaningful cohort of underrepresented patients to the existing literature. Future prospective, multi-institutional studies with larger sample sizes and comprehensive chemotherapy data are needed to validate these findings and further elucidate the relationship between KRAS mutation status, ethnic variation in tumor genomics, and outcomes following HIPEC.

This study must be evaluated in the context of its limitations. As a single-institution, retrospective analysis with a relatively small sample size—particularly in the wildtype KRAS cohort (n = 28)—statistical power is limited, increasing the possibility of type II error. Although baseline characteristics were generally comparable between groups, clinically important differences in prognostic factors such as preoperative PCI and completeness of cytoreduction were observed despite not reaching statistical significance. The limited sample size precluded multivariable regression, and residual confounding cannot be excluded. Additionally, detailed neoadjuvant chemotherapy data, including the number of cycles and lines of therapy, were not available for analysis, as many patients were referred from outside institutions with incomplete treatment records. Although no statistically significant difference in outcomes was observed between KRAS-mutant and wildtype patients who received neoadjuvant chemotherapy, the absence of granular chemotherapy data limits our ability to assess the impact of treatment duration on outcomes.

## 5. Conclusions

In this study evaluating the efficacy of CRS/HIPEC for treatment of advanced-stage colon cancer with peritoneal metastases, we found no differences in recurrence-free survival between patients with mutant versus wildtype *KRAS* mutations. We also found no differences in perioperative complications, ICU and hospital length of stay, and postoperative PCI. While *KRAS* mutations have previously been reported as poor prognostic indicators for patients with advanced-stage colorectal cancer, our findings suggest the opposite and imply *KRAS* mutations may not be poor prognostic indicators for inferior survival in metastatic colon cancer patients with peritoneal metastases undergoing CRS/HIPEC. We show in this study that patients with *KRAS* mutations have comparable recurrence-free survival compared to patients with wildtype *KRAS* when treated with CRS/HIPEC. These findings may imply that CRS/HIPEC offers therapeutic benefits for *KRAS*-mutant patients, potentially mitigating the usually adverse prognosis associated with this mutation. These findings also suggest that mutant-type *KRAS* may be an important selection factor to consider in optimizing patient selection for CRS/HIPEC.

## Figures and Tables

**Table 1:** Patient sociodemographic and tumor characteristics.

		Wildtype	Mutant *KRAS*	Total	*p*-Value
		n (%)	n (%)	n (%)	
**Age**	Median (IQR)	48 (44, 60)	53 (45, 63)	52 (44, 61)	0.335
**Gender**	Female	15 (53.6%)	33 (63.5%)	48 (60.0%)	0.389
	Male	13 (46.4%)	19 (36.5%)	32 (40.0%)	
**Race**	Asian	0 (0.0%)	1 (1.9%)	1 (1.3%)	0.628
	Black	5 (17.9%)	7 (13.5%)	12 (15.0%)	
	Other	0 (0.0%)	3 (5.8%)	3 (3.8%)	
	Unknown	1 (3.6%)	1 (1.9%)	2 (2.5%)	
	White	22 (78.6%)	40 (76.9%)	62 (77.5%)	
**Ethnicity**	Hispanic or Latino	15 (53.6%)	30 (57.7%)	45 (56.3%)	0.384
	Not Hispanic or Latino	12 (42.9%)	22 (42.3%)	34 (42.5%)	
	Missing	1 (3.6%)	0 (0.0%)	1 (1.3%)	
**Preoperative ECOG Performance Status**	0	12 (42.9%)	19 (36.5%)	31 (38.8%)	0.146
	1	6 (21.4%)	19 (36.5%)	25 (31.3%)	
	2	0 (0.0%)	5 (9.6%)	5 (6.3%)	
	3	1 (3.6%)	1 (1.9%)	2 (2.5%)	
	Missing	9 (32.1%)	8 (15.4%)	17 (21.3%)	
**Symptoms at Presentation**	Mild (pain, small ascites)	15 (53.6%)	29 (55.8%)	44 (55.0%)	0.873
	None	3 (10.7%)	4 (7.7%)	7 (8.8%)	
	Severe (obstruction)	8 (28.6%)	17 (32.7%)	25 (31.3%)	
	Missing	2 (7.1%)	2 (3.8%)	4 (5.0%)	
**Clinical Stage at Diagnosis**	Stage II	1 (3.6%)	3 (5.8%)	4 (5.0%)	0.573
	Stage III	6 (21.4%)	16 (30.8%)	22 (27.5%)	
	Stage IV	21 (75.0%)	33 (63.5%)	54 (67.5%)	
**Tumor Grade**	I	1 (3.6%)	3 (5.8%)	4 (5.0%)	0.575
	II	15 (53.6%)	31 (59.6%)	46 (57.5%)	
	III	7 (25.0%)	14 (26.9%)	21 (26.3%)	
	Not Available	5 (17.9%)	4 (7.7%)	9 (11.3%)	
**Mucinous Histology on Pathology**	No	17 (60.7%)	29 (55.8%)	46 (57.5%)	0.149
	Yes	8 (28.6%)	22 (42.3%)	30 (37.5%)	
	Missing	3 (10.7%)	1 (1.9%)	4 (5.0%)	
**Oncologic Surgery prior to CRS/HIPEC**	No	10 (35.7%)	25 (48.1%)	35 (43.8%)	
	Yes	18 (64.3%)	27 (51.9%)	45 (56.3%)	0.288
**No. of Oncologic Surgeries prior to CRSHIPEC**	Median (IQR)	1 (1, 2)	1 (1, 2)	1 (1, 2)	0.262
**Neoadjuvant** **Chemotherapy**	No	8 (28.6%)	24 (46.2%)	32 (40.0%)	
	Yes	20 (71.4%)	28 (53.8%)	48 (60.0%)	0.126
**Surgery and NAC** **prior to CRS/HIPEC**	No	10 (35.7%)	25 (48.1%)	35 (43.8%)	
	Yes	18 (64.3%)	27 (51.9%)	45 (56.3%)	0.288
**Time (months)** **Diagnosis to CRSHIPEC**	Median (IQR)	19 (11, 40)	23 (14, 43)	20 (12, 42)	0.478

**Table 2:** Operative factors and postoperative outcomes.

		Wildtype	Mutant *KRAS*	Total	*p*-Value
		n (%)	n (%)	n (%)	
**PCI at Exploration**	Median (IQR)	7 (3, 12)	11 (5, 21)	8 (4, 18)	0.057
**Agent Used During HIPEC**	Cisplatin	1 (3.6%)	1 (1.9%)	2 (2.5%)	
	Mitomycin C	26 (92.9%)	51 (98.1%)	77 (96.3%)	0.349
	Oxaliplatin	1 (3.6%)	0 (0.0%)	1 (1.3%)	
**Number of Anastomoses Performed**	Median (IQR)	1 (1, 2)	1 (1, 2)	1 (1, 2)	0.818
**Number of Organs Debulked**	Median (IQR)	4 (3, 6)	5 (4, 8)	5 (4, 8)	0.224
**Number of Peritonectomies**	Median (IQR)	1 (0, 3)	2 (0, 4)	2 (0, 4)	0.645
**Ascites Drained During HIPEC**	No	22 (78.6%)	39 (75.0%)	61 (76.3%)	
	Yes	2 (7.1%)	4 (7.7%)	6 (7.5%)	0.932
	Not Available	4 (14.3%)	9 (17.3%)	13 (16.3%)	
**Resection Score**	R0	22 (84.6%)	33 (68.8%)	55 (74.3%)	
	R1	3 (11.5%)	7 (14.6%)	10 (13.5%)	0.323
	R2a	0 (0.0%)	3 (6.3%)	3 (4.1%)	
	R2b	1 (3.8%)	1 (2.1%)	2 (2.7%)	
	R2c	0 (0.0%)	4 (8.3%)	4 (5.4%)	
**Postoperative PCI**	Median (IQR)	0 (0, 0)	0 (0, 0)	0 (0, 0)	0.055
**Hospital Length of Stay**	Median (IQR)	9 (7, 12)	10 (7, 15)	10 (7, 14)	0.256
**ICU Length of Stay**	Median (IQR)	3 (2, 6)	3 (2, 5)	3 (2, 6)	0.803
**Clavien-Dindo Classification**	0	4 (14.3%)	1 (1.9%)	5 (6.3%)	**0.015**
	1	18 (64.3%)	23 (44.2%)	41 (51.3%)	
	2	4 (14.3%)	16 (30.8%)	20 (25.0%)	
	3 or more	2 (7.1%)	12 (23.1%)	14 (17.5%)	
**Status of Patient at Last Known Follow-Up**	Alive with Disease	10 (35.7%)	19 (36.5%)	29 (36.3%)	0.515
	Dead of Disease	11 (39.3%)	21 (40.4%)	32 (40.0%)	
	Dead of Other Cause	1 (3.6%)	1 (1.9%)	2 (2.5%)	
	No Disease	6 (21.4%)	8 (15.4%)	14 (17.5%)	

## Data Availability

Data are unavailable due to privacy restrictions.
